# The Influence of Baseline Marijuana Use on Treatment of Cocaine Dependence: Application of an Informative-Priors Bayesian Approach

**DOI:** 10.3389/fpsyt.2012.00092

**Published:** 2012-10-30

**Authors:** Charles Green, Joy Schmitz, Jan Lindsay, Claudia Pedroza, Scott Lane, Rob Agnelli, Kimberley Kjome, F. Gerard Moeller

**Affiliations:** ^1^University of Texas Health Sciences Center at HoustonHouston, TX, USA; ^2^SAS, Inc.Cary, NC, USA

**Keywords:** cocaine, marijuana, treatment response, Bayesian, subgroup analysis

## Abstract

**Background:** Marijuana use is prevalent among patients with cocaine dependence and often non-exclusionary in clinical trials of potential cocaine medications. The dual-focus of this study was to (1) examine the moderating effect of baseline marijuana use on response to treatment with levodopa/carbidopa for cocaine dependence; and (2) apply an informative-priors, Bayesian approach for estimating the probability of a subgroup-by-treatment interaction effect. **Method:** A secondary data analysis of two previously published, double-blind, randomized controlled trials provided complete data for the historical (Study 1: *N* = 64 placebo), and current (Study 2: *N* = 113) data sets. Negative binomial regression evaluated Treatment Effectiveness Scores (TES) as a function of medication condition (levodopa/carbidopa, placebo), baseline marijuana use (days in past 30), and their interaction. **Results:** Bayesian analysis indicated that there was a 96% chance that baseline marijuana use predicts differential response to treatment with levodopa/carbidopa. Simple effects indicated that among participants receiving levodopa/carbidopa the probability that baseline marijuana confers harm in terms of reducing TES was 0.981; whereas the probability that marijuana confers harm within the placebo condition was 0.163. For every additional day of marijuana use reported at baseline, participants in the levodopa/carbidopa condition demonstrated a 5.4% decrease in TES; while participants in the placebo condition demonstrated a 4.9% increase in TES. **Conclusion:** The potential moderating effect of marijuana on cocaine treatment response should be considered in future trial designs. Applying Bayesian subgroup analysis proved informative in characterizing this patient-treatment interaction effect.

## Introduction

Multiple substance use is common in cocaine patients, making it a challenge to obtain samples of “pure” or singly dependent subjects for clinical trials research. The response has been to broaden the eligible subject pool by allowing concurrent use or abuse of other substances, although recent articles have debated the degree of acceptable heterogeneity for pharmacotherapy efficacy research (O’Brien and Lynch, 2003; Rounsaville et al., 2003). Moreover, when patients who use multiple drugs are included in a cocaine clinical trial, the investigation needs to take into account this increased variability. Not all clinical trials have consistently reported the impact of concomitant substance use on response to cocaine treatment.

Marijuana use is especially prevalent among patients with cocaine dependence (Miller et al., 1990a,b; Aharonovich et al., 2005, 2006). In an earlier report our research group described some interesting characteristics associated with marijuana-using individuals presenting for medication treatment trials (Lindsay et al., 2009). Among the large percentage (46.4%) of concurrent marijuana users seeking treatment for cocaine dependence, those who used marijuana frequently (i.e., 10 or more days over the past 30) showed a profile of greater clinical impairment, exemplified by more severe addiction severity scores, and heavier patterns of drug use, than those who used marijuana occasionally or not at all.

The extent to which marijuana-using cocaine-dependent patients fare worse in treatment compared to their non-marijuana-using counterparts is unclear. Aharonovich et al. (2006) examined the impact of continued cannabis use during methylphenidate treatment of comorbid cocaine dependence and ADHD and found that moderate or intermittent use of cannabis was associated with better retention in treatment than heavy use or abstinence. The effect of cannabis use on cocaine abstinence rates was not significant, contrary to previous findings (Aharonovich et al., 2005). Other studies have failed to find an effect of marijuana use on retention or cocaine treatment outcomes (Budney et al., 1996; Higgins et al., 2003). In a recent retrospective analysis of data from three clinical trials of contingency management, Alessi et al. (2011) found that pretreatment marijuana use did not dampen the benefit of this behavioral treatment in reducing cocaine use. In sum, the small literature shows mixed results on the impact of cannabis use on cocaine treatment outcome, underscoring the need to better understand this potential interaction, especially as it relates to the development of effective medications for treatment of cocaine dependence.

The prognostic significance of baseline marijuana use on treatment outcome is the focus of the present study. Having reported positive results from earlier trials of levodopa pharmacotherapy for cocaine dependence (Mooney et al., 2007; Schmitz et al., 2008), we carried out a secondary analysis to determine whether levodopa treatment effects varied as a function of marijuana use at baseline. Observations by Lindsay et al. (2009) led us to expect that marijuana use would moderate the effect of levodopa by reducing overall treatment effectiveness. Specifically, we hypothesized that within the subgroup of cocaine subjects having high levels of baseline marijuana use, treatment with levodopa pharmacotherapy would be less effective.

The second aim of this study is to demonstrate a more appropriate, statistical method for conducting secondary analysis of subgroup treatment effects. Analytically, evaluation of subgroup effects entails testing the interaction of treatment and some baseline, subgroup measurement. A large literature has criticized the use of traditional, Frequentist approaches as inadequate due to their often low power, and dichotomous evaluation of the evidence (i.e., significance testing; Dixon and Simon, 1991; Simon et al., 1996; Simon and Freedman, 1997; Simon, 2002; Green et al., 2009). For instance, Brookes et al. (2001) showed that a trial with 80% power to detect a main effect of treatment possessed only 29% power to detect an interaction effect as large as the main effect. Positing an interaction effect matching the magnitude of a main effect reflects an extremely optimistic scenario. Stated another way, if the interaction was as large as the anticipated main effect, the sample size would still require quadrupling in order to maintain 80% power to detect the interaction. The same literature, criticizing conventional Frequentist methods for analyzing treatment-by-subgroup interaction effects, has recommended Bayesian methods as an alternative.

Bayesian analytical methods avoid issues of reduced power and significance testing by evaluating the probability that the alternative (H_A_) hypothesis exists, given the observed data, and any prior evidence for H_A_. These probabilities can be meaningfully refined, unlike *p* values that cannot, by using informative prior probability distributions (for more extensive review of the limitations of *p* values, and the advantages of Bayesian statistical approaches see Dixon and Simon, 1991; Schervish, 1996; Goodman, 1999, 2005; Berry, 2006). Thus, the researcher is able to attach a probability value to the parameter of interest, in this case, the interaction effect, e.g., “given the observed data, the probability that an interaction of at least magnitude *X* exists is *Y*.” The Bayesian approach provides a natural analysis for the present situation, in which we sought to incorporate information from a previous study to improve precision in estimating the interaction parameter. Referred to as the Bayesian *power prior* approach, this paper demonstrates how to pool historical data from an earlier randomized clinical trial with current data, and produce more precise conclusions regarding the hypothesis of interest, i.e., that heterogeneity in response to treatment is a function of baseline marijuana use.

## Materials and Methods

### Sample

The present study analyzed data obtained from two completed levodopa trials for cocaine dependence. In Study 1, cocaine-dependent subjects enrolled in a 9-week, randomized, double-blind, placebo-controlled trial comparing placebo to 400/100 mg and 800/200 mg levodopa/carbidopa in a sustained-release preparation (Sinemet CR). In Study 2, cocaine-dependent subjects received levodopa/carbidopa (800/200 mg) or placebo delivered in combination with behavioral therapy interventions of varying intensity. Study 1 established the safety, tolerability, and feasibility of levodopa therapy in cocaine outpatients, while Study 2 demonstrated support for use of levodopa pharmacotherapy with behavioral contingency management. Details of each trial design, sample composition, and main findings are presented elsewhere (Mooney et al., 2007; Schmitz et al., 2008). The samples used here are subsets of the overall trial data, selected due to their comparability in dosing for levodopa (800/200 mg), and behavioral intervention (cognitive-behavioral therapy without contingency management). To demonstrate the proposed Bayesian analysis, Study 1 (*N* = 64 complete observations) formed the historical dataset and Study 2 (*N* = 113 complete observations) provided the current dataset.

All participants provided written informed consent. The Committee for the Protection of Human Subjects (CPHS) of the University of Texas Medical School, Houston reviewed and approved the research, consent, and all study materials.

### Measures

#### Moderator variable

Baseline level of marijuana use was defined according to self-reported number of days of use over the past 30 using the Addiction Severity Index (ASI; McLellan et al., 1980). In addition to self-reported marijuana use, urine specimens were screened for Δ-9-THC, the primary active metabolite of marijuana. The concordance rate between self-report and urinalysis testing was acceptable (76%).

#### Treatment response variable

The Treatment Effectiveness Score (TES; Ling et al., 1997) was used as an outcome indicator of treatment response. The TES is calculated by assigning one point for each cocaine-negative urine sample (cutoff < 300 ng/ml), and no points for positive or missing samples. Both studies required three urine samples per week. Studies 1 and 2 were 9 and 12 weeks respectively permitting TES values that ranged from 0–27 to 0–36 negative urines.

### Analysis

#### Bayesian statistical reasoning

Frequentist and Bayesian statistical reasoning comprise the principal modes of statistical reasoning, taking distinct but symbiotic approaches to uncertainty/probability. Frequentist reasoning defines probability as the frequency of an event in the limit of a series of infinite, repeated trials. Often illustrated using a large number of repeated coin flips, counting the number of heads, or tails, long run frequency counts provide an estimate of the fixed parameter that is governs the performance of the coin. Bayesian reasoning defines probability as a judgment regarding the likelihood of an event (Hájek, 2003). Often referred to as a subjective or personalist probability, Bayesian reasoning is best operationalized in terms of the proportion of a fixed amount of money an observer would bet on a specific outcome (Hájek, 2003). Statistically, the Frequentist approach models the data as random, and the parameter as fixed and unknown. In contrast the Bayesian approach models a parameter as unknown and random, and the observed data as fixed (Lucke, 2004). Frequentist reasoning indirectly evaluates the alternative hypothesis (H_A_) by rejecting or failing to reject the null hypothesis (H_0_). This permits statements such as “given that the null hypothesis is true, the probability of observing data this extreme or more extreme is *Z*.” Indirect evaluation of the H_A_ by testing H_0_ means that no probability valuation is attached to directly to possible values of H_A_. While Frequentist Confidence Intervals represent an attempt at providing an index of H_A_ they are properly interpreted as those interval which have a 95% chance of capturing the true parameter estimate and say nothing about the differential plausibility of the values they include. They cannot, because in Frequentist statistics the true parameter estimate is fixed (i.e., it has a probability of one): while the true parameter is fixed, the confidence interval moves with each new sample. Bayesian reasoning, defines the governing parameter of interest (in this case the interaction coefficient) as random, and therefore permits probability statements regarding what the value of that parameter might be. The product of a Bayesian analysis is the posterior distribution, which indicates the differential probability that the parameter of interest takes various values. Since the relative probability of various values being the true parameter within this interval is defined by the shape of the posterior distribution, it is possible to comment on the probability that H_A_ takes on some value or range of values. This means that, given the observed data and a formally articulated prior distribution representing the anticipated value and uncertainty for the parameter of interest, Bayesian modeling permits direct quantification of evidence for the alternative hypothesis (H_A_). The Bayesian approach permits statements such as “given the observed data, the probability that an interaction of at least magnitude *X* exists is *Y*.” Under certain conditions (e.g., vague priors) the values of the estimates from Bayesian and Frequentist methods are often quite similar, however their interpretation is quite different: Bayesian inference directly addresses the alternative hypothesis, while Frequentist inference does so indirectly by rejecting or failing to reject the null hypothesis. In making this distinction the current paper is not arguing that one approach is inherently correct and the other not. Rather, we agree with Kendall (1949) that each of these different notions of probability ultimately cannot stand without the other. More recently, Wijeysundera et al. (2009) pointing out that the approaches answer different albeit complementary, questions.

#### Bayesian posterior probabilities

In this paper we present a Bayesian analyses as an alternative approach to assessing the association between baseline marijuana use and treatment effectiveness. In brief, Bayesian reasoning holds that inferences about a hypothesis should be encapsulated in a probability distribution, given the observed data. This distribution, known as the posterior probability distribution, summarizes evidence for the parameter value as the product of previous evidence (prior distribution), and any newly gathered data. Bayes’ Theorem (Eq. 1) expresses this relation:

(1) $$p\left(\theta |\text{data}\right)=\frac{p\left(\text{data}|\theta \right)p\left(\theta \right)}{p\left(\text{data}\right)}$$

where *p*(data|θ) is the likelihood and *p*(θ) is the prior distribution. The denominator *p*(data) functions as a scaling coefficient and is often omitted from the equation to give:

(2) $$p\left(\theta |\text{data}\right)\propto p\left(\text{data}|\theta \right)p\left(\theta \right)$$

Specifically, the probability that the parameter takes on some value (or range of values) is proportional to the product of the observed data (i.e., likelihood) and prior evidence. Stated in prose, this reads, “The probability (i.e., “*p*”) of the parameter value θ given (i.e., “|”) the data is proportional to (i.e., “∝”) the probability of the data given the parameter value θ [i.e., “*p*(data| θ)”] multiplied by the prior probability of θ [i.e., “*p*(θ)”].

#### Bayesian prior distributions

The prior distribution is a mathematical formalization of existing evidence for a parameter value before observing new data (Gill, 2002). In the absence of historical evidence, the prior distribution is based on subjective judgment, representing varying degrees of skepticism regarding the pre-existing evidence (i.e., enthusiastic, neutral, or skeptical). When access to data from previous studies is available, it is scientifically reasonable and statistically advantageous to incorporate this historical information into the prior distribution. However this requires investigators to evaluate the comparability or *exchangeability* of the historical and current data. Ibrahim and Chen advocate the use of *power priors* (Chen et al., 2000; Ibrahim and Chen, 2000; Ibrahim et al., 2003; Chen and Ibrahim, 2006) to produce posterior distributions that incorporate historical data sets. Implementation of such methods permits the evaluation of the sensitivity of posterior estimates to assumptions regarding the comparability of the constituent samples. Assuming that such comparability exists, combining the information existing in more than one sample may have the benefit of improving the precision of the resulting estimates.

#### Bayesian power priors

Study 1 provided the existing, historical data set (*D*_0_ of size *n*_0_) and Study 2 provided the current data set (*D*_1_ of size *n*_1_). We assumed an initial prior distribution (i.e., before observing *D*_0_) represented by *p*(θ). Incorporation of *D*_0_ into the estimation of the posterior distribution based on *D*_1_ takes the following form:

(3) $$p\left(\theta |D_{1}\right)\propto p\left(D_{1}|\theta \right)p{\left(D_{0}|\theta \right)}^{a_{0}}p\left(\theta \right)$$

Where $p{\left(D_{0}|\theta \right)}^{a_{0}}p\left(\theta \right)$ represents the power prior which incorporates the historical data from *D*_0_, raised to the power *a*_0_ which is restricted to 0 ≤ *a*_0_ ≤ 1, and the initial prior [i.e., *p*(θ)]. Conceptually, values of *a*_0_ range from *a*_0_ = 0, in which *D*_0_ is fully discounted or excluded from the model, to *a*_0_ = 1 in which *D*_0_ carries the same weight as *D*_1_. In essence *a*_0_ provides a means of regulating the amount of information that *D*_0_ contributes to the analysis of *D*_1_. Two reasons for adjusting the weight of the historical data are: (1) discounting very large historical data sets so that they do not overwhelm the information in a smaller, more current data set, and (2) accounting for inter-sample heterogeneity which may result from differences in the experimental protocol, sampling, etc. (Chen and Ibrahim, 2006; De Santis, 2006). While empirical estimation of *a*_0_ from the data might reflect a weighting that minimizes the loss of information across the two samples (Ibrahim et al., 2003; Chen and Ibrahim, 2006) such an approach is problematic (Neuenschwander et al., 2009), leading to extremely low estimates of *a*_0_ even for identical data sets (Neelon and O’Malley, 2010). Moreover, an attempt to remedy this (Duan et al., 2005) leads to improved but low estimates of *a*_0_ (Neelon and O’Malley, 2010). We adopt a “conditional power priors” (Neelon and O’Malley, 2010, p. 2) approach, following Spiegelhalter et al. (2004) and evaluating the full range of values for *a*_0_ to understand the degree to which discounting the historical data influences inferences based on the resulting posterior distribution.

#### Model specification

Negative binomial regression evaluated TES as a function of treatment condition (levodopa/carbidopa, placebo), baseline marijuana use (number of days in the past 30), and their interaction. Unlike Poisson regression, negative binomial regression accounts for over-dispersion in the count data characterizing both the historical and current data sets. Following Eq. 3, initial prior distributions [i.e., *p*(θ)] took the form ∼Normal (0, var = 1 × 10^6^) for regression coefficients (i.e., normally distributed with a mean of zero and a variance of 1,000,000; the prior is centered on the null hypothesis and expresses substantial uncertainty). While SAS 9.3 specifies a ∼Gamma(0.001, 0.001) prior as a default for the dispersion term (Gamma functions describe a family of distribution on the positive real number line. The parameters values 0.001 and 0.001 refer to as the α and β parameters respectively. These two quantities capture the shape (i.e., α) and rate (i.e., β) of the specified distribution. The inverse of the rate (i.e., 1/β) is called the scale parameter. The two parameters convey the shape and the spread or dispersion of the distribution. In, perhaps more familiar terms, the mean of a Gamma distributed variable is α/β while its variance is α/β^2^). There has been some concern that such priors may not be appropriate (see Gelman, 2006 for a discussion in the context of normal distributed data). As such we follow DiPrete et al. (2011) as well as Zheng et al. (2006). Reasoning that the dispersion parameter might take values ranging (0–∞), these authors specify a ∼Uniform (0,1) prior on the inverse of the dispersion coefficient [a ∼Uniform (0,1) distribution spans the range zero through one with all values having an equal probability of occurring (i.e., it is a straight line)]. SAS v. 9.3 code for doing so is relatively straightforward and available from the corresponding author. These vague, neutral prior distributions acknowledge a relative state of ignorance regarding parameter values prior to observing either the historical or current data sets. Again, following Eq. 3, the likelihood incorporating the historical data (i.e., $p{\left(D_{0}|\theta \right)}^{a_{0}}$) required specification of the term *a*_0_. Following Spiegelhalter et al. (2004) we evaluated use of the historical data at values ranging from *a*_0_ = 0 to *a*_0_ = 1. Estimates of parameter values for the interaction term are the appropriate indices for evaluating heterogeneity in response to treatment as a function of baseline marijuana use. As in Poisson regression, exponentiated parameter estimates and intervals correspond to risk ratios (R.R.) and Credible Intervals (C.B.I.) respectively.

#### Computational software

Analyses utilized Proc Markov Chain Monte Carlo (MCMC; SAS v. 9.3; SAS Institute Inc, 2010) which provides a flexible computing environment for the MCMC simulations for estimating Bayesian posterior distributions. An example applying Proc MCMC to a relatively straightforward use of power priors in estimating a binomial proportion using historical and current data is provided in SAS Documentation (SAS Institute Inc, 2011). Examples of statistical code and salient output for the current analysis are available from the corresponding author.

## Results

Baseline marijuana use characteristics of pharmacotherapy groups from their respective studies are provided in Table 1.

**Table 1:** **Baseline marijuana use levels**.

Variable	Placebo	l-DOPA
	Mean, SD (*N*)	Mean, SD (*N*)
**HISTORICAL DATA**		
Baseline marijuana use (days in past 30)	3.41, 7.68 (29)	1.97, 5.67 (35)
Baseline marijuana use (years of use)	9.17, 10.06 (29)	11.03, 9.96 (34)
**CURRENT DATA**		
Baseline marijuana use (days in past 30)	3.28, 7.23 (58)	3.24, 6.94 (55)
Baseline marijuana use (years of use)	13.43, 10.71 (58)	10.55, 9.21 (55)

**Figure 1:**
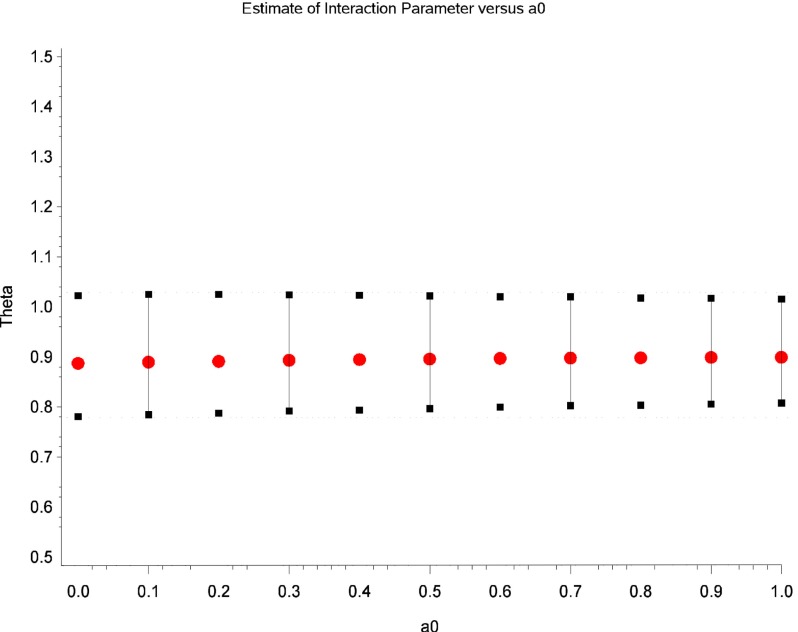
**Estimates of the parameter for the interaction term (i.e., theta) as a function of different values of *a*_0_**.

**Figure 2:**
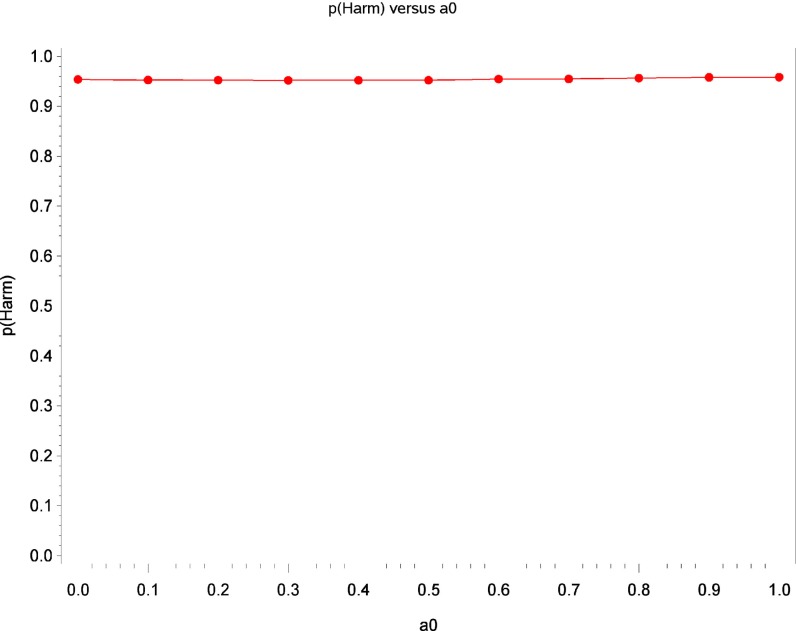
**Estimates of the probability that increased baseline marijuana use predicts decreased TES scores in the levodopa treatment as a function of *a*_0_**.

**Figure 3:**
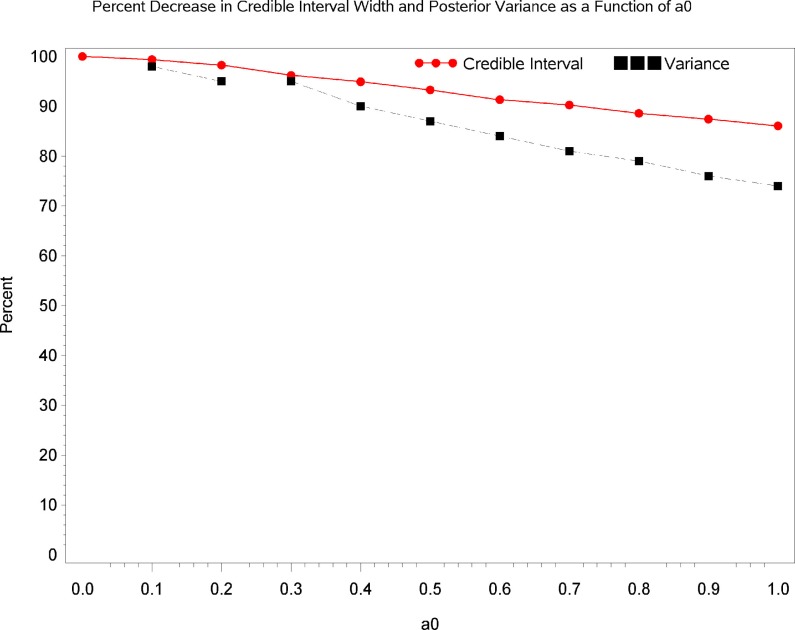
**Change in the precision of the parameter estimate as a function of *a*_0_**. The red line shows the magnitude of the decrease in the Credible Interval range as a percentage of its width at *a*_0_ = 0, as a function of altering *a*_0_. The blue line shows the magnitude of the decrease in the variance of the posterior distribution as a function of altering *a*_0_.

**Figure 4:**
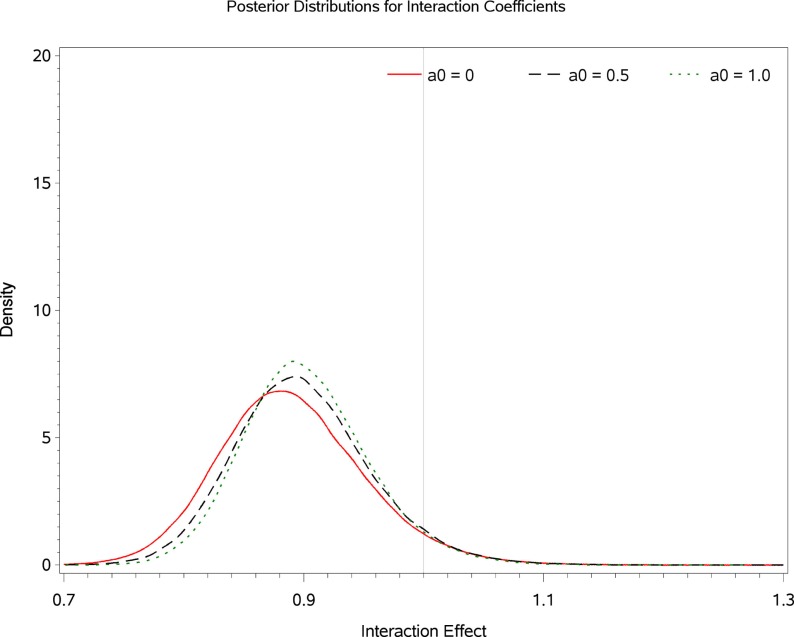
**Posterior distributions of the interaction parameter at values of *a*_0_ = 0, 0.5, and 1.0**.

Table 2 displays Bayesian estimates for *a*_0_ = 0 and *a*_0_ = 1 as well as frequentist estimates of the interaction term. Estimates of the interaction coefficient for *a*_0_ = 0 and *a*_0_ = 1 and *a*_0_ = 1 are R.R. = 0.887 (95% C.B.I. 0.781–1.022) and R.R. = 0.899 (95% C.B.I. 0.808–1.016) respectively. That is, relative to patients in the placebo condition, participants receiving levodopa demonstrated a decrease in TES by a factor of 0.887 (i.e., 11.3%) or 0.899 (i.e., 10.1%) for the two most extreme weights that might be attached to the historical evidence (i.e., *a*_0_ = 0 and *a*_0_ = 1 respectively). Note that the point estimates are quite similar and that the 95% Credible Interval for *a*_0_ = 0 entirely contains the corresponding interval for *a*_0_ = 1. The broader credible interval for *a*_0_ = 0 which encompass the interval for *a*_0_ = 1 reflects the decrease in uncertainty that occurs as the historical data receives greater weight in the analysis; a reasonable result given that inclusion of more information from the historical data permits greater precision in the resulting estimates. Inspection of Figures 1 and 2 show that, at different levels of *a*_0_, the value of the parameter estimate for the interaction, as well as the probability that increased baseline marijuana use predicts decreased TES scores in the levodopa condition, remains relatively constant. Inspection of Figure 3 shows the magnitude of the decrease in the Credible Interval range and the variance of the posterior distribution as a percentage of its width at *a*_0_ = 0, as a function of altering *a*_0_. In the current case, the range of the Credible Interval decreases by approximately 14% while the variance of the posterior distribution of the exponentiated coefficient decreases by almost 26%. Finally, Figure 4 shows the posterior distributions of the parameter value for the interaction coefficient. The distributions, displayed for *a*_0_ = 0, 0.5, and 1.0, appear to be quite similar. Given the failure to demonstrate that alterations in *a*_0_ result in substantial changes to the estimated value of the interaction coefficient, coupled with the increase in precision for the estimate, it is reasonable to give the historical data a weight comparable to the current data (i.e., *a*_0_ = 1).

**Table 2:** **Exponentiated parameter estimates and 95% credible intervals for *a*_0_ = 0 and *a*_0_ = 1**.

	Risk ratio	95% Credible limits	
		95% Lower limit	95% Upper limit
**NEGATIVE BINOMIAL REGRESSION FOR TES**			
***a**_**0**_ = **0***
Intercept	3.158	1.895	5.694
Medication	3.346	1.483	7.650
Marijuana	1.062	1.000	1.160
Medication*marijuana	0.887	0.781	1.022
Dispersion	28.566	11.451	107.651
***a**_**0**_ = **1***
Intercept	3.376	2.184	5.552
Medication	2.376	1.217	4.646
Marijuana	1.050	0.999	1.124
Medication*marijuana	0.899	0.808	1.016
Dispersion	38.695	16.805	118.570
**FREQUENTIST MAXIMUM LIKELIHOOD ESTIMATES FOR THE INTERACTION OF MEDICATION AND MARIJUANA**			
**Sample**	**Risk ratio estimate**	**95% Confidence interval**	**Likelihood ratio statistic**

Historical sample	0.856	0.687–1.066	χ(1) = 1.77, *p* < 0.184
Current sample	0.887	0.787–1.001	χ(1) = 3.12, *p* < 0.078
Combined samples	0.899	0.810–0.998	χ(1) = 3.30, *p* < 0.069

From a substantive perspective, using *a*_0_ = 1, the probability that baseline marijuana use predicts differential response to treatment with levodopa/carbidopa is 0.96 (i.e., where θ is less than or equal to a R.R. of 1.0; Figure 4). Based on the Frequentist Likelihood Ratio Test statistics presented in Table 1, neither the historical, current nor the combined data sets lead to rejection of the null hypothesis.

**Figure 5:**
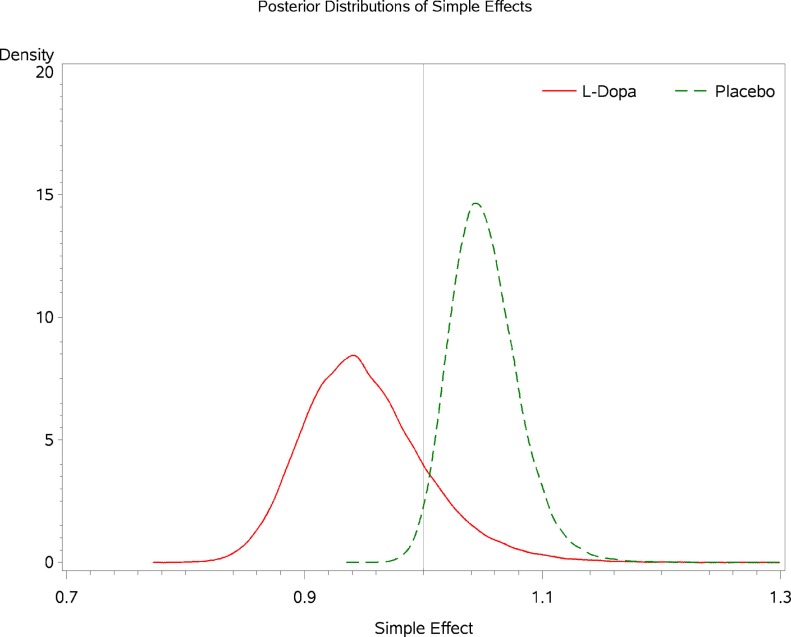
**TES as a function of baseline marijuana and treatment condition**.

**Figure 6:**
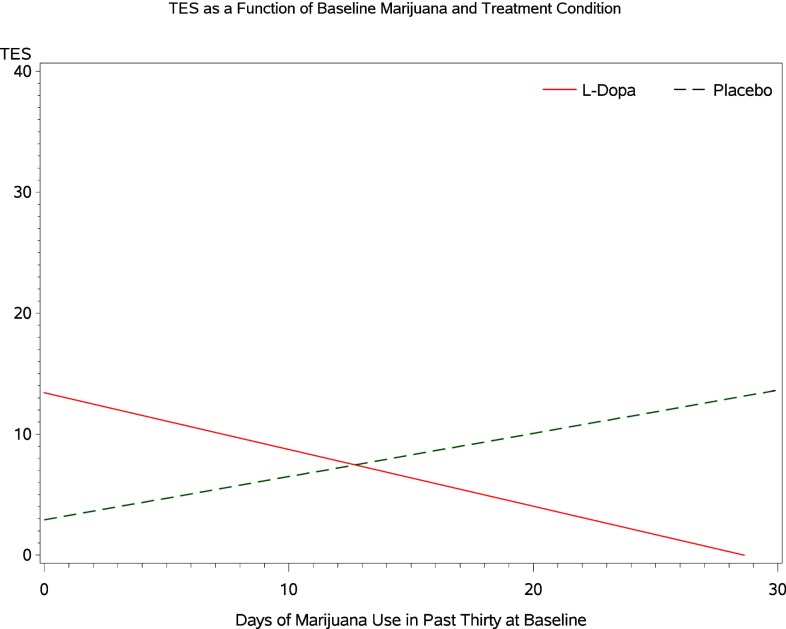
**Posterior densities for the simple effect of baseline marijuana use within each condition**.

Inspecting the simple effects of baseline marijuana use indicates that among participants receiving levodopa/carbidopa the probability that baseline marijuana confers harm in terms of treatment outcome is 0.981 (Figure 5). The probability that marijuana confers harm within the placebo condition is 0.163 (Figure 5). For every additional day of marijuana use reported at baseline, participants in the levodopa/carbidopa condition demonstrate a 5.4% decrease in TES (R.R. 0.946, 95% C.B.I. 0.864–1.069; Figure 6). For every additional day of marijuana use reported at baseline, participants in the placebo condition demonstrate a 4.9% increase in TES (R.R. 1.049, 95% C.B.I. 1.002–1.115; Figure 6).

## Discussion

Subgroup analyses are informative for characterizing heterogeneity in response to treatment for cocaine dependence. Inadequate power minimizes the usefulness of conventional, Frequentist tests of the salient interaction terms. As such Bayesian methods provide a more appropriate, probabilistic measure of the evidence for subgroup effects. Improved precision for estimates of subgroup effects can result from incorporation of historical data into the analysis of a current data set. Doing so raises questions regarding the comparability of the data sets, and the weight subsequently accorded the historical data. Power priors offer a mechanism for formalizing the degree to which historical data is incorporated into the analysis of a newer data set.

In the current context this type of analysis points, with high probability, to the existence of a subgroup effect of baseline marijuana use on response to treatment for cocaine dependence with levodopa/carbidopa. Specifically, higher marijuana use predicted lower treatment effectiveness (cocaine-negative urines) in the levodopa/carbidopa condition, but not in the placebo condition. It is possible that higher marijuana use at baseline operates as a proxy for cocaine severity that has been shown to distinguish subpopulations in terms of treatment response. Repeating our analysis with baseline cocaine use as a covariate, however, failed to alter any substantive conclusions. To the extent that frequent marijuana use at baseline continued during treatment, this pattern of concomitant use may have influenced levodopa’s efficacy via providing competing drug reinforcement, perhaps counteracting the putative dopamine-restoring effects of levodopa. Although generally a well-tolerated medication in cocaine-dependent patients, levodopa may interact with marijuana to produce less tolerable effects and thus reduce compliance and efficacy. Based on these findings, future research should examine how marijuana use *during* treatment interacts with cocaine use in moderating the effects of levodopa treatment: (i) chronic marijuana use may produce changes in cannabinoid-1 and/or dopamine receptor availability which may alter the effects of levodopa in cocaine-dependent subjects; (ii) intoxication following acute marijuana use may alter the probability of concurrent cocaine use in the levodopa condition relative to placebo via drug–drug interactions not yet fully understood.

Limitations of the current study include the *post hoc*/secondary nature of the data analysis. A large literature has discussed the problem of *post hoc* subgroup analyses (Adams, 1998; Assmann et al., 2000; Pocock et al., 2002; Cook et al., 2004; Rothwell, 2005). The clear danger is that *post hoc* selection of subgroups for analysis may result in capitalization on chance variability in the data. While this is somewhat mitigated by the finding that the interaction remains consistent, pooling across the two samples, it is still possible that idiosyncrasies in recruitment, or secular trends in the population from which sampling occurred resulted in biased estimates of the interaction effect. Prospective confirmation of these findings have broad implications for analyzing patient heterogeneity in response to treatment, the design of clinical trials to account for population heterogeneity through stratified randomization, as well as more specific implications for the potential usefulness of levodopa pharmacotherapy in treatment cocaine dependence.

## Conflict of Interest Statement

Robert Agnelli who was instrumental in programming the SAS statistical code is an employee of SAS, Inc., Cary, NC, USA. The other authors conducted the current research in the absence of any commercial or financial relationships that could be construed as a potential conflict of interest.
